# Interplay of stromal tumor-infiltrating lymphocytes, normal colonic mucosa, cancer-associated fibroblasts, clinicopathological data and the immunoregulatory molecules of patients diagnosed with colorectal cancer

**DOI:** 10.1007/s00262-021-02863-1

**Published:** 2021-02-24

**Authors:** Łukasz Zadka, Mariusz Chabowski, Damian Grybowski, Aleksandra Piotrowska, Piotr Dzięgiel

**Affiliations:** 1 Department of Human Morphology and Embryology, Division of Histology and Embryology, Chałubińskiego 6a, 50-368 Wrocław, Poland; 2 Department of Clinical Proceedings, Faculty of Health Science, Wroclaw Medical University, Wroclaw, Poland; 3 Department of Surgery, 4Th Military Teaching Hospital, Wroclaw, Poland; 4 Department of Surgery, University of Chicago, Chicago, USA

**Keywords:** Colorectal cancer, Tumor infiltrating lymphocytes, Cancer-associated fibroblasts, Ki-67, PD-L1, CD14

## Abstract

A total of 94 patients with colorectal cancer (CRC) were included in this study. Lymphocytic infiltration of CD45^+^ cells in the normal colon was more pronounced than that in the paired tumor stroma (*p* = 0.0008). The mean immunoscore of CD45^+^TILs was decreased in CRC compared with the controls (*p* = 0.0010). The percentage of CD3^+^ cells was higher in stage II than in stage IV (*p* = 0.0218) and showed a negative correlation with the TNM classification (r = -0.2867, *p* = 0.0109). The number of stromal CD4^+^TILs was higher in stage I than in stage III (*p* = 0.0116) and IV (*p* = 0.0104), and there was a negative correlation between this number and the stage (r = -0.3708, *p* = 0.0008). There was a positive correlation between the Ki-67 and CD45^+^ (r = 0.2468, *p* = 0.0294), CD3^+^ (r = 0.3822, *p* = 0.0006), and CD4^+^ cells (r = 0.5465, *p* < 0.0001). The levels of cancer-associated fibroblast (CAF) markers such as α-SMA, thrombin and fibronectin were significantly higher in CRC than in normal colonic mucosa. The immunohistochemical expression of α-SMA was negatively correlated with TILs, while fibronectin showed positive coexpression. A higher number of cells expressing IL-2Rα, PD-L1, CD33 and CD14 were found in colorectal adenocarcinomas than in controls. The number of CD14^+^ cells was also dependent on the TNM stage (*p* = 0.0444) and tumor budding (*p* = 0.0324). These findings suggest a suppressive impact of CRC on the adaptive immune response and emphasize the importance of CAFs in regulating tumor immunity.

## Introduction

Colorectal cancer (CRC) is the third most commonly diagnosed neoplasm worldwide and is one of the leading causes of death among oncological patients for which the incidence and mortality rates increase significantly with age [1]. Progressive deterioration of the immune system that occurs with aging may remain an important trigger in the development of many tumors [2]. On the other hand, some components of immune response related to cellular immunity and the secretory functions of the immune cells may lead to oncogenesis. Chronic inflammation of the colonic mucosa and ongoing molecular-structural remodeling during its duration from a long-term perspective are key factors in the development of CRC; thus, inflammation is part of a well-defined pathological pathway for this type of cancer [1, 3]. In addition, more attention is currently being given to the factors affecting the distribution of inflammatory cells in the tumor milieu. The components of the extracellular matrix (ECM), through their specific biochemical properties, can regulate the migration of immune cells toward the development of cancer. Intratumoral fibrosis, whose production is mostly dependent on cancer-associated fibroblasts (CAFs), seems to be of particular interest [4]. CAFs can produce numerous ECM components that mediate the regulation of tumor immunity. On the other hand, CAFs, as part of the tumor microenvironment, may be a useful biomarker in the molecular phenotyping of tumors due to their high heterogeneity [5]. The best known CAF markers include alpha smooth muscle actin (α-SMA), collagen, fibroblast activation protein (FAP) and fibronectin [6]. Fibroblast activation protein (FAP) is a widespread biomarker of CAFs associated with both TME remodeling and an unfavorable prognosis. Tumor-associated fibroblasts expressing FAP also show immunosuppressive potential against the TME, and the molecule itself is considered a new molecular target in cancer therapy [5]. A major role is also played by thrombin, which directly stimulates the proliferation of fibroblasts and the synthesis of ECM components [4]. This phenomenon may indirectly affect the distribution of tumor infiltrating lymphocytes (TILs) by limiting the migration of immune cells to the tumor nest.

A separate topic comprises biomarkers with direct effects on the immune response. Recently, the important role of interleukin-2 (IL-2) signaling in the pathogenesis of CRC has been underlined. This cytokine, in addition to the activation of T cells, may increase the risk of CRC development and has prognostic significance in this type of cancer [7]. Moreover, the increased preoperative level of serum soluble interleukin-2 receptor (IL-2R) in patients with colorectal cancer may suggest receptor release by activated inflammatory cells in the tumor milieu [8]. Activation of the IL2/IL-2R axis is associated with the JAK-STAT signaling pathway, which may be related to cancer development as well as potential applications in cancer immunotherapy [9–11]. JAK-STAT signaling is also associated with tumor-associated macrophages (TAMs), which are an independent indicator of a poor prognosis in various cancers and can accelerate the development of CRC by remodeling the ECM and affecting CAF activity [12, 13]. Cluster of differentiation 14 (CD14) is an important marker of macrophages that also serves important regulatory functions for monocytes when in soluble form [14]. Programmed cell death ligand-1 (PD-L1) is another crucial immune regulator in CRC that is actively released by both cancer and inflammatory cells and leads to T cell exhaustion, which has prognostic value [15, 16]. The suppressive activity of inflammation is also regulated by the CD33 molecule, whose expression is specific for both myeloid and some subtypes of immune cells [17].

The secretory immunosuppressive activity of cancer cells can interfere with antitumor immunity, which leads to changes in cellular responses as well as prognosis. An imbalance between TH1 and TH2 subsets of CD4^+^ T cells with a tendency for immunological polarization toward the TH2 phenotype has been confirmed in peripheral venous blood in patients diagnosed with CRC and may be associated with the progression of this cancer [18]. The cellular immune response in CRC associated with the presence of effector memory T cells showed a correlation with the histopathological traits of cancer and revealed significant prognostic value [19].

A frequently raised issue is the possibility of using TILs in the diagnosis of selected cancers and in monitoring the response to implemented cancer immunotherapy. Regarding CRC, high TIL densities were associated with better survival rates among treated patients and a significant reduction in mortality [20]. In stage III CRC, the assessment of TILs using standard hematoxylin and eosin staining in combination with estimation of the neutrophil-to-lymphocyte ratio (NLR) was a sufficient solution that improved the prognosis among patients who underwent surgery followed by adjuvant FOLFOX chemotherapy [21]. The location of TILs in the tumor stroma or in the tumor nest showed a relationship with both histopathological data and selected clinical parameters of CRC which confirmed its importance [22]. The value of TILs in CRC in predicting survival seemed to even exceed that of the widely used UICC-TNM classification which is necessary to determine the stage of the disease [23].

The current trend is to assess the importance of TILs in relation to known markers routinely used in histopathology. Ki-67 is a cellular proliferation antigen with high diagnostic and prognostic value in many cancer types. Its increased expression ​​is most often associated with an unfavorable prognosis, including in CRC [24]. Nevertheless, there are well-documented reports of the beneficial importance of a high Ki-67 index in CRC. High immunohistochemical expression of Ki-67 is a favorable prognostic factor in CRC [25] In turn, Li et al. confirmed the favorable prognostic value of Ki-67 in stage III and IV CRC [26]. The relationship between TILs and the Ki-67 index in CRC has not yet been assessed.

In this study, we assessed the correlation between immunohistochemical coexpression of TIL markers and Ki-67. Moreover, the difference between the intensity of inflammatory infiltrates in the normal colonic mucosa and number of TILs has been estimated as well as the relationship between the TILs and TNM stage, histopathological grade and selected clinicopathological data. In an additional comparison of inflammatory infiltrates between the normal colonic mucosa and tumor tissue, we used matched pairs of samples taken from individual patients. We also evaluated the coexpression of TIL and CAF markers. Independent assessments were conducted for immunoregulatory molecules such as CD14, IL-2Rα, CD33 and PD-L1. Immunohistochemical coexpression of these markers highlights the immunosuppressive activity of CRC. To the best of our knowledge, this is the first study estimating these variables.

## Materials and methods

### Patients

Formalin-fixed, paraffin-embedded (FFPE) blocks taken from 107 patients with colorectal adenocarcinoma were included in this study. Tissue fragments were collected intraoperatively during resection of the tumor between 2013 and 2016 from patients who underwent colectomy procedures in the Department of Surgery of the 4th Military Teaching Hospital in Wroclaw. Diagnoses were made based on the expertise of a qualified pathologist. None of the patients who qualified for the study had previously undergone radio or chemotherapy as a form of systemic treatment. The exclusion criteria were as follows: use of immunosuppressive drugs in the three months before tumor resection; any incident of inflammatory bowel disease/autoimmune disease; and any other neoplasms in the patient’s past medical history. Healthy surgical margins as a valid control prior to inclusion in the study were evaluated microscopically on standard histological slides. The presence of cancer cells excluded the sample from further analysis. To ensure the most homogeneous group of CRC, all patients included in the study were selected with possibly similar clinical data. From the 107 paraffin blocks included in the study 78 cases of CRC samples and healthy colonic mucosa surgical margins (*n* = 29) were separated for further analysis. A separated analysis was carried out by selecting all matched pairs of CRC tissue and cancer-associated healthy colonic mucosa taken from individual patients (*n* = 13). Tumor infiltrating lymphocytes (TILs) were identified based on the immunohistochemical expression of CD45, CD3, CD4 and CD8 antigens in 78 CRC paraffin blocks. Only lymphocytic infiltrates situated in the tumor stroma were taken into consideration. The exact characteristics of patients corresponding to the samples within which stromal TILs were assessed are presented in table (Table 1).

**Table 1:** Clinicopathological characteristic of patients diagnosed with colorectal adenocarcinoma (*n* = 94)

Tissue type of the tested sample	Colorectal cancer (CRC)	Healthy colonic mucosa	Cancer-associated colonic mucosa (matched pairs)
*N*	78	29	13/29
Age (years)			
Minimum	42	50	52
Maximum	88	88	86
Mediam	71	69	75
Sex			
Female	36	14	8
Male	42	15	5
TNM stage		n/a	n/a
I	12		
II	29		
III	23		
IV	14		
T parameter		n/a	n/a
Tx	0		
T0	0		
Tis	0		
T1	1		
T2	16		
T3	57		
T4	4		
(T1 + T2)	17		
(T3 + T4)	61		
Tumor grade (G)		n/a	n/a
G1	26		
G2	46		
G3	6		
Anatomical site of the primary lesion (colon)			
Ascending	20	4	3
Transverse	6	4	2
Descending	3	4	0
Sigmoid	27	7	5
Rectum	22	10	3
Histopathological type of adenocarcinoma		n/a	n/a
Mucinous	12		
Non-mucinous	66		

N/A Not applicable

### Tissue microarrays

Tissue microarrays (TMAs) were created with the use of the TMA Grand Master (3DHistech, Budapest, Hungary) automatic tissue microarrayer. From each paraffin block, 6-μm-thick sections were cut and stained with H&E. Next, the prepared slides were scanned with a Pannoramic MIDI II (3DHistech) histological scanner. Representative spots for the TMAs (3 spots 1.5 mm in diameter from each paraffin block) were selected by a qualified pathologist from the digital slides with the use of CaseViewer (3DHistech) software.

### Immunohistochemistry (IHC)

Immunohistochmical reactions were performed on 4-µm-thick slides from the TMA. Initially, slides were deparaffined and rehydrated and then antigen retrieval was carried out by boiling the sections in EnVision FLEX Target Retrieval Solution pH 9 (CD3, CD4, CD8, CD14, CD45, α-SMA, fibronectin, IL-2Rα) or pH 6 (Ki-67, PD-L1) using a PTLink system (Dako, Glostrup, Denmark). The activity of endogenous peroxidase was blocked for 5 min. with EnVision FLEX Peroxidase-Blocking Reagent (Dako). Mouse monoclonal antibodies against CD45 (IR751, clone 2B11 + PD7/26), CD4 (IR649, clone 4B12), CD8 (IR623, clone C8/144B), Ki-67 (IR626, clone MIB-1), CD14 [4B4F12] (ab182032, abcam), CD33 (1:200 dilution; orb153436, Biorbyt), PD-L1 (1:100 dilution; M3653; Dako), thrombin (1:100 dilution; thrombin antibody F-1, sc-271449, Santa Cruz Biotechnology), and α-SMA (RTU, IR611, Dako) and rabbit polyclonal antibodies against CD3 (IR503, Dako, RTU), fibronectin (1:400 dilution; NBP1-91,258, Novusbio), IL-2 receptor alpha (EPR6452, 1:100 dilution; ab128955, Abcam), type III collagen (1:200 dilution; ab7778, Abcam) and rabbit monoclonal antibodies against FAP-α (EPR20021, 1:250 dilution; ab207178, Abcam) were used as primary antibodies (20 min.). Then, slides were incubated with EnVision FLEX/HRP (20 min.). The reactions were visualized with freshly prepared 3,3′-diaminobenzidine (DAB) (10 min.). Additionally, slides were stained with EnVision FLEX Hematoxylin (5 min.). After the immunohistochemical reactions and counterstaining, slides were dehydrated in ethanol (70%, 96%, absolute) and xylene and then mounted with Dako mounting medium (Dako). Immunohistochemical reactions were performed on an Autostainer Link 48 system (Dako). Digital slides were examined using CaseViewer (3DHistech) software.

### Semi-quantitative scoring system

**Fig. 1:**
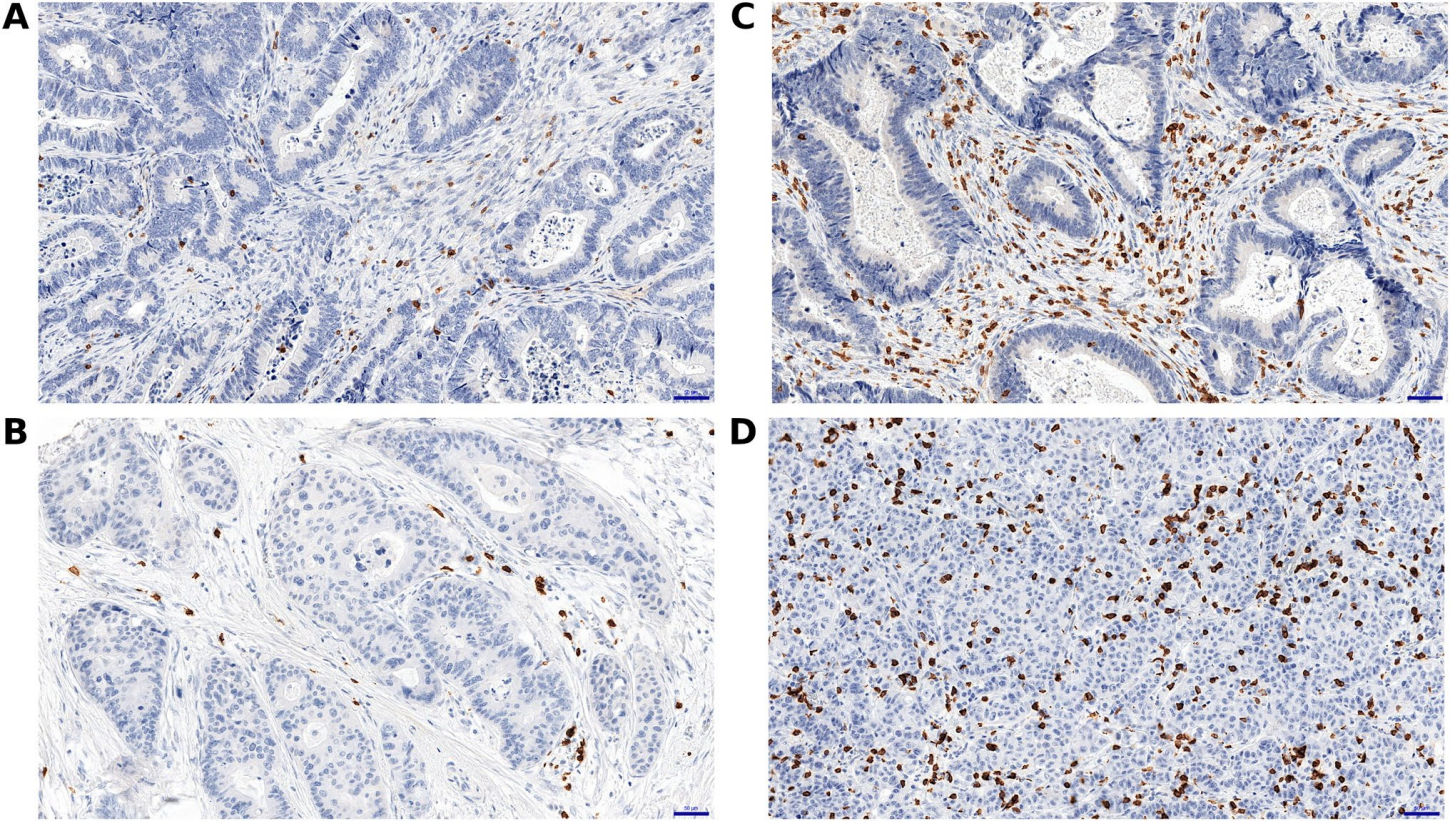
Representative immunohistochemical staining of tumor infiltrating lymphocytes (TILs) in tumor stroma. **a** CD3^+^ cells; 1-point **b** CD8^+^ cells; 1-point **c** CD3^+^ cells, 3-points **d** CD8^+^ cells, 3-points. Scale bar = 50 μm

In the assessment of the immunohistochemical reactions, a modified method based on the Remmele and Stegner scoring system was used, referred to as the “immunoreactive score” [27]. The percentage of all positive cells present in the connective tissue compartment or tumor stroma was evaluated as follows: no positive reaction (0 points); < 10% positive cells (1 point); 10 – 50% positive cells (2 points); 51 – 80% positive cells (3 points) and > 80% of positive cells (4 points). TILs were evaluated in the tumor stroma in accordance with the International TILs Working Group (ITWG) system methodology [28]. The only exceptions to the adopted recommendations were TIL evaluation by the hotspot method in the examined TMA tissue fragments. Moreover, in the TIL evaluation we used IHC for immunostaining and not the recommended method of TIL assessment on standard histological slides for hematoxylin and eosin staining. For each case, three of the most representative matrix spots were selected for further examinations. Two independent researchers evaluated the cases included in the study (LZ, AP). The obtained percentages were reviewed and verified by a qualified pathologist (PD). Representative immunohistochemical reactions and scores are presented graphically (Fig. 1). CAF markers were determined based on the positive reactions against α-SMA, type III collagen, thrombin, fibroblast activation protein, alpha (FAP-α) and fibronectin. Due to the noticeable differences in the distribution of positive reactions against fibronectin (FN) and thrombin for connective tissue and epithelial cells, the expression levels of these markers were assessed independently for both epithelial cells and the lamina propria of the colonic mucosa of healthy surgical margins and then compared to the corresponding tumor tissue. The same analyses were performed for selected immunoregulatory molecules such as CD14, IL-2Rα, CD33 and PD-L1. TMAs for these reactions were estimated by the hotspot method. In the assessment of the tissue distribution of α-SMA expression, the vascular beds, muscularis mucosae and muscularis externa layers were excluded from the analyses due to the natural expression of this biomarker on smooth muscle cells [29]. This exclusion criterion was applied to both healthy colonic mucosa and adenocarcinomas. Due to the observed tissue distribution of α-SMA, collagen and FAP-α in the connective tissue, the differences between the CRC and control samples were assessed only in the tumor stroma and for the corresponding normal colonic mucosa.

## Tumor budding assessment in colorectal cancer

Microscopic clusters of undifferentiated cancer cells at the invasive front of the tumor, otherwise defined as “tumor budding,” were evaluated in accordance with the International Tumor Budding Consensus Conference (ITBCC) 2016 recommendations [30]. In our study, the TMA cores for standard staining by hematoxylin and eosin were used to evaluate this parameter. The slides were examined using an Olympus BX41 light microscope (Olympus, Tokyo, Japan) at 20 × magnification. Each tissue fragment was evaluated by the hotspot method (hotspot, 0.785 mm2). The following scoring system was used for each core: 0 – 4 buds (Bd1, low); 5 –9 buds (Bd2, intermediate); and ≥ 10 buds (Bd3, high).

## Assessment of additional histopathological parameters of colorectal adenocarcinomas

The proliferation grade of the examined tumors was established based on previously performed immunohistochemical staining against Ki-67 and the adopted method of the semiquantitative evaluation of positive reactions. Neoplastic lesions of the colon were classified as having low proliferating if the expression level of Ki-67 did not exceed 10% of all positive tumor cells. Moderate proliferating adenocarcinomas were determined in the range of 10–50% and tumors with more than 50% of the positive tumor cells against Ki-67 were defined as highly proliferating lesions. The assignment of the studied cancers with the appropriate anatomical location enabling their subclassification into left-sided and right-sided CRC was carried out in accordance with the assumptions of the American Society of Clinical Oncology (ASCO), defining left-sided CRC as lesions of the splenic flexure and in regions distal to the splenic flexure, including rectal tumors [31]. Right-sided CRC as defined by the ASCO included lesions of the cecum and the ascending colon up to the hepatic flexure. All primary tumors located in the transverse colon were excluded from further analyses, including the assessment of the influence of the anatomical site on the expression of the studied markers.

## Multicolor digital detection of examined antigens for chromogenic IHC staining

**Fig. 2:**
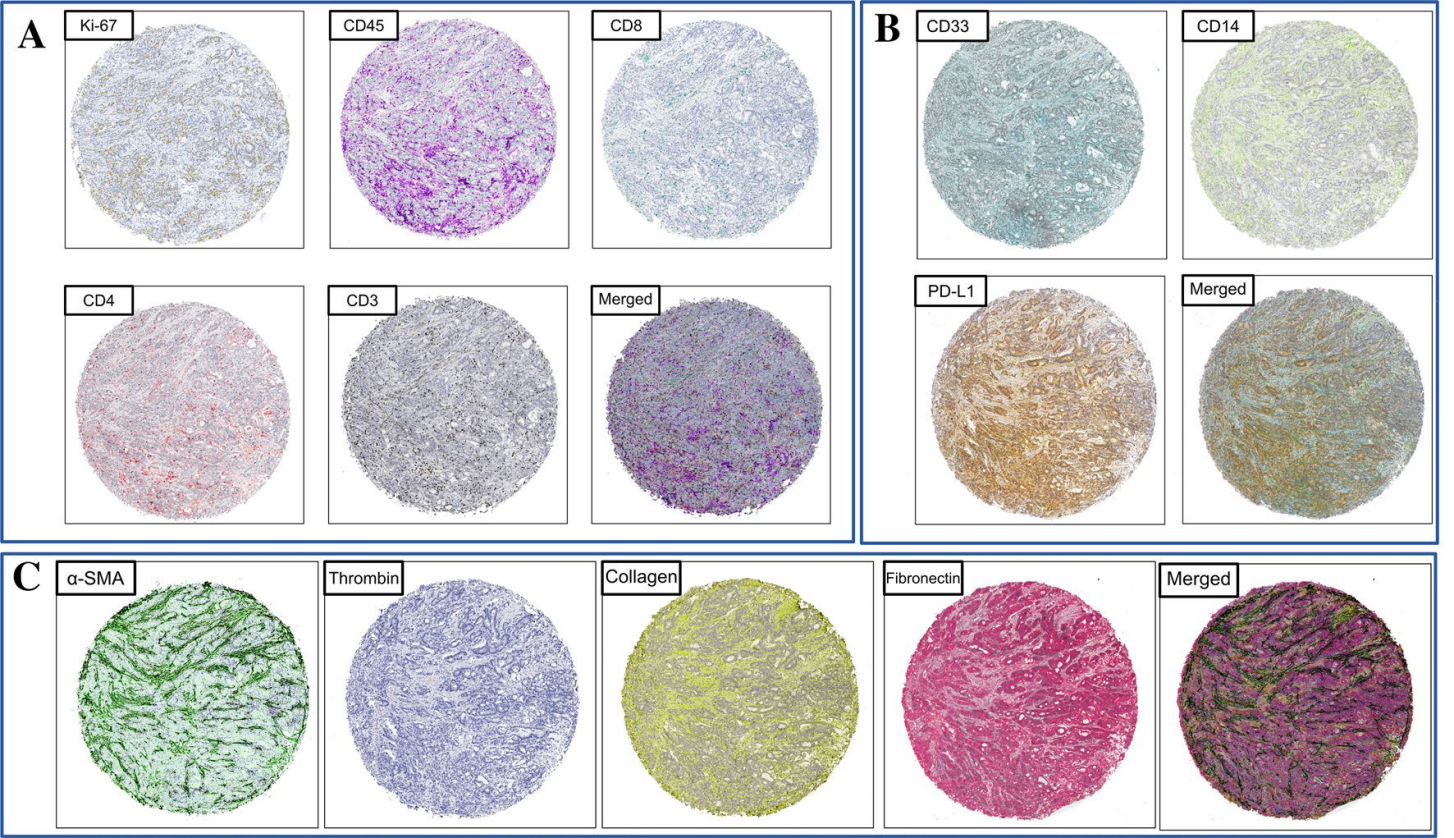
Digital multicolor colocalization assessment on serial sectioning of chromogenic IHC of tested markers for; **a** TILs and Ki-67 **b** immunoregulatory molecules; **c** CAFs. Visualization of the method on the example of representative TMAs core; human colorectal adenocarcinoma

Digital visualization of the colocalization of multiple tested markers was performed with GIMP software (ver. 2.10.8). Distinct TMA punches were rotated to the same angle and centered. Then, the dark-brown color of the immunohistochemical reaction was changed with the “Hue-Saturation” tool to distinct colors, which were different for each antigen. Then, all layers’ modes were changed to “darken only.” The resulting merged image contained colors assigned for all antigens. Figure 2 presents merged images, as well as their component parts (Fig. 2).

## Statistical analysis

Statistical evaluation of the obtained results was carried out using GraphPad Prism 8.1.2 software (332). The selection of an appropriate test was preceded in each case by an assessment of the normality of the distribution. The unpaired t test was used to analyze the differences in the expression levels of the investigated markers between CRC and healthy controls. To assess the difference between inflammatory infiltrates in the normal control tissue and the number of TILs in the corresponding synchronous CRC, the paired t test was used. One-way Anova together with Tukey’s multiple comparison test was used to examine the relationship between the TIL immunophenotype, tumor grade and stage of CRC. In assessing the relationship between the T parameter of the TNM classification and TILs, the unpaired t test was used due to significant disproportions in the number of groups for T1 and T4, and hence, only two groups were further evaluated (T1 + T2 vs T3 + T4). The difference in CAFs and immunoregulatory molecules expression according to selected histopathological parameters was estimated by Kruskal–Wallis (for multiple group comparisons) and Mann–Whitney tests (for two group comparisons). Correlations and their significance were calculated using the Pearson test. P values < 0.05 (two-tailed) were considered statistically significant.

## Results

The number of CD45^+^ positive cells was significantly decreased in tumor stroma compared to healthy surgical margins.

**Fig. 3:**
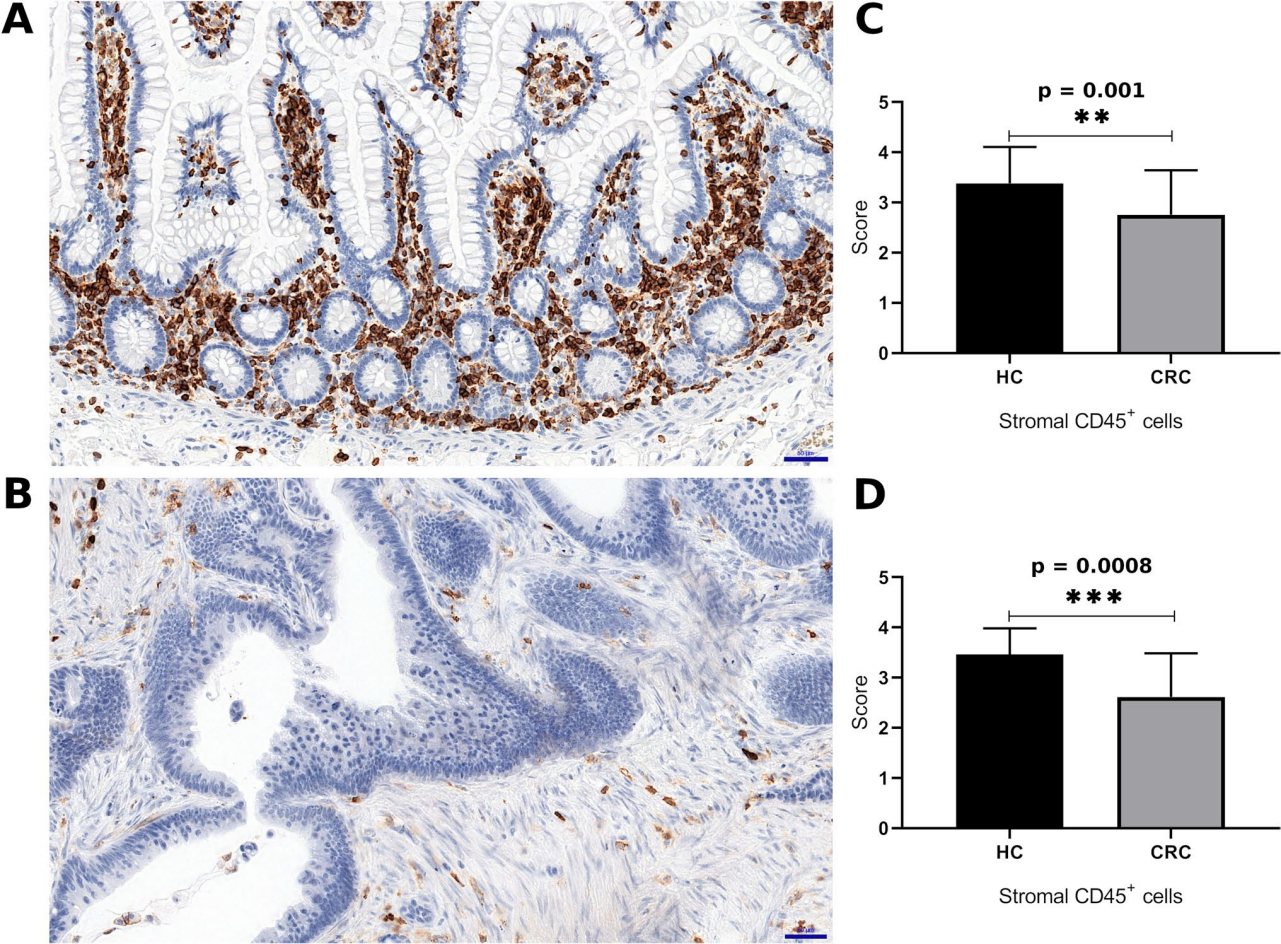
Comparison of inflammatory infiltrations intensity between normal colonic mucosa (**a**) and colorectal cancer (**b**). **c** Histogram shows the percentage of CD45 +  cells in healthy surgical margins (HC; *n* = 29) and colorectal cancer (CRC; n = 78 cases). **d** Estimation of immunohistochemical expression of CD45 antigen, matched pairs (*n* = 13). In both cases, the following scoring system was used: no positive reaction (0 points); < 10% positive cells (1 point); 10%–50% positive cells (2 points); 51%–80% positive cells (3 points); and > 80% of positive cells (4 points). Scale bars = 50 μm

The immunohistochemical assessment of CD45 antigen performed in 107 cases showed a differentiated intensity of inflammatory cells between the tumor stroma (*n* = 78) and normal control (*n* = 29). Significantly lower expression of CD45 was found in CRC than in the healthy margins (*p* = 0.0010) (Fig. 3c). Due to the disproportion in the abundance between the CRC cohort and healthy controls (HC), an additional analysis of samples associated with each other was performed using matched pairs of samples from individual patients. In the assessment of 13 pairs of HC and associated tumor sites, the paired t test showed a significantly higher intensity of inflammatory infiltrates in the HC tissue than in the CRC cases, which confirmed the results obtained previously (p = 0.0008) (Fig. 3d). In each case of a single positive immune cell, a clear specific membrane reaction was present. A specific membrane reaction was relatively often accompanied by less pronounced coexpression of CD45 in the cytoplasm, and the intensity of the cytoplasmic reaction was clearly higher in TILs than in inflammatory cells in the control samples. No significant differences were found in the expression of CD3^+^, CD4^+^ and CD8^+^ cells, by IHC between HC and CRC (p = 0.1754, p = 0.1764, p = 0.7778, respectively).

There was a positive correlation between TILs and the cellular proliferation index in CRC.

**Fig. 4:**
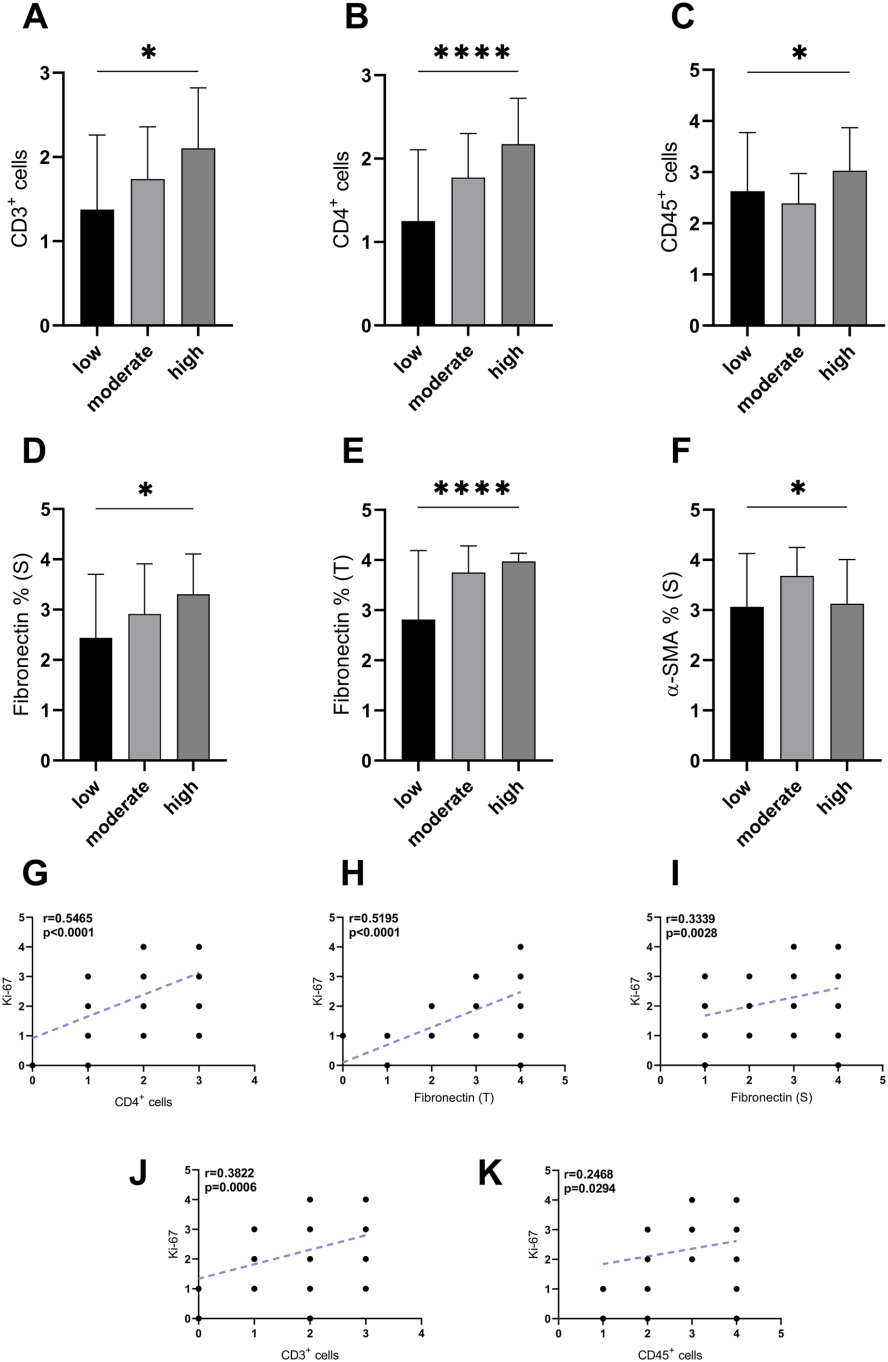
**a–c** Relationship between the tumor proliferation grade and the percentage level of stromal tumor infiltrating lymphocytes (TILs); **d–f** cancer-associated fibroblasts (CAFs) markers. Correlations between Ki-67 and both TILs (**g**, **j**, **k**) and fibronectin (**h**, **i**) in patients diagnosed with colorectal cancer, *p* < 0.05 (*); *p* < 0.0001 (****); correlations were performed with the Pearson test

MIB-1 is a monoclonal antibody against Ki-67, which is a well-known marker of cellular proliferation and is correlated with neoplastic invasion and progression [32–34]. In the assessment of the coexpression of individual TIL markers and Ki-67, a tendency for a positive correlation was found. CD45^+^ TILs showed a weak positive correlation with the cellular proliferation antigen (r = 0.2468, p = 0.0294), while an even stronger trend was observed in the range of helper T cell markers. In regard to CD3^+^ TILs, moderately strong positive correlation with Ki-67 was noted (r = 0.3822, p = 0.0006); however, a strong positive correlation was found between CD4^+^ cells and Ki-67 (r = 0.5465, p < 0.0001). There was a tendency for a weak positive correlation between CD8^+^ cells and Ki-67 (r = 0.1020); however, no statistical significance was obtained (p = 0.3740) (Fig. 4).

The TNM stage affects the stromal TIL immunophenotype.

Relevant CRC stages were determined using the assumptions and principles of the latest (8th edition) American Joint Committee on Cancer TNM classification [35, 36]. CRC cases classified as T1/T2/N0/M0 were included in stage I. Stage II included all patients with parameters corresponding to stages IIA, IIB and IIC. Stage III included all patients with features corresponding to IIIA, IIIB and IIIC, whereas stage IV included cases of CRC with distant metastasis.

A negative correlation was found between the CRC stage (I-IV) and number of TILs. The Pearson correlation test showed statistical significance for CD3^+^ TILs (r = -0.2867, p = 0.0109) and for CD4^+^ TILs (r = -0.3708, p = 0.0008). CD8^+^ and CD45^+^ cells showed a weak negative correlation with stromal TILs; nevertheless, the obtained results were not statistically significant (r = -0.1337, p = 0.2431; r = -0.1142, p = 0.3197, respectively).

In some stages, there was also a significant difference in the intensity of inflammatory infiltrates. ANOVA (I—IV) showed statistical significance in terms of the percentage of CD3^+^ cells (p = 0.0336) obtained in the TILs between stages II and IV (p = 0.0218). More pronounced statistical significance was noted for CD4^+^ TILs (p = 0.0066) by differentiating inflammatory infiltrates between stages I and III (p = 0.0116) and IV (p = 0.0104). For CD45^+^/CD8^+^ TILs, no statistically significant results were obtained (Table 2). Due to the significant prognostic value of T parameters and the recently debated value of N in determining the CRC stage [37, 38], it was decided to perform an independent analysis determining the relation of these features to TILs. The T parameter showed a statistically significant weak negative correlation with CD4^+^ TIL (r = -0.2775, p = 0.0139), while the number of involved lymph nodes (pN) did not significantly correlate with the stromal TIL immunophenotype (Table 2).

**Table 2:** Stromal tumor-infiltrating lymphocytes (TILs) in colorectal cancer and its association with clinicopathological data and immune regulatory molecules

Variables	Immune cells			
	CD3^+^	CD4^+^	CD8^+^	CD45^+^
	r (Pearson test)			
	*p* value (2-tailed)			
TNM stage	*r* = −0.2867	*r* = −0.3708	*r* = −0.1337	*r* = −0.1142
	*p* = 0.0822	*p* = 0.0008	*p* = 0.0179	*p* = 0.0130
	*p* = 0.0336	*p* = 0.0066	*p* = 0.5546	*p* = 0.0972
pT	*r* = −0.0856	*r* = −0.2775	*r* = −0.0068	*r* = −0.1224
	*p* = 0.4559	*p* = 0.0139	*p* = 0.9527	*p* = 0.2855
pN	*r* = 0.0408	*r* = −0.0267	*r* = 0.1116	*r* = 0.0875
	*p* = 0.7230	*p* = 0.8167	*p* = 0.3092	*p* = 0.4460
Tumor grading	*r* = −0.1741	*r* = −0.1135	*r* = 0.1159	*r* = −0.0465
	*p* = 0.1275	*p* = 0.3226	*p* = 0.3121	*p* = 0.6862
	*p* = 0.1212	*p* = 0.2360	*p* = 0.3386	*p* = 0.6862
Demographic data				
Age	*r* = 0.014	*r* = −0.122	*r* = 0.162	*r* = 0.228
	*p* = 0.903	*p* = 0.288	*p* = 0.156	*p* = 0.045
Sex (Female/Male)	*p* = 0.3038	*p* = 0.6425	*p* = 0.4046	*p* = 0.4249
Anatomical site of primary CRC				
Left-sided	*p* = 0.3784	*p* = 0.6112	*p* = 0.1338	*p* = 0.7458
Right-sided				
Histopathology				
Mucinous	*p* = 0.9794	*p* = 0.6748	*p* > 0.9999	*p* = 0.3688
Non-mucinous				
Macroscopic subtype				
Ulcerated	*p* = 0.1693	*p* = 0.0152	*p* = 0.3698	*p* = 0.9482
Non-ulcerated				
Tumor size ≤ 2 cm & > 2 cm	*p* = 0.9843	*p* = 0.5237	*p* = 0.0711	*p* = 0.3661
Tumor budding	*p* = 0.3038	*p* = 0.6425	*p* = 0.4046	*p* = 0.4249
Immune regulators				
Expression pattern				
Stromal				
PD-L1	*r* = 0.320	*r* = 0.316	*r* = −0.036	*r* = 0.172
	*p* = 0.0045	*p* = 0.005	*p* = 0.7566	*p* = 0.1340
CD14	*r* = 0.243	*r* = 0.389	*r* = −0.048	*r* = 0.102
	*p* = 0.03308	*p* = 0.00047	*p* = 0.6758	*p* = 0.3794
CD33	*r* = 0.292	*r* = 0.127	*r* = −0.090	*r* = 0.216
	*p* = 0.0096	*p* = 0.2686	*p* = 0.4308	*p* = 0.0576
IL-2Rα	*r* = 0.245	*r* = 0.093	*r* = 0.256	*r* = 0.331
	*p* = 0.032	*p* = 0.423	*p* = 0.025	*p* = 0.003
Tumoral				
PD-L1	*r* = 0.264	*r* = 0.408	*r* = −0.160	*r* = 0.166
	*p* = 0.02	*p* = 0.0002	*p* = 0.1632	*p* = 0.1488
CD14	*r* = 0.116	*r* = 0.371	*r* = 0.014	*r* = −0.006
	*p* = 0.315	*p* = 0.001	*p* = 0.901	*p* = 0.958
CD33	*r* = 0.327	*r* = 0.102	*r* = 0.050	*r* = 0.134
	*p* = 0.003	*p* = 0.376	*p* = 0.662	*p* = 0.244
IL-2Rα	*r* = 0.164	*r* = 0.132	*r* = 0.481	*r* = 0.378
	*p* = 0.155	*p* = 0.254	*p* < 0.0001	*p* = 0.001

*TNM* tumor–node–metastasis classification, *CRC* colorectal cancer, *PD-L1* Programmed cell death ligand-1, *CD14* Cluster of differentiation 14, *IL-2Rα* interleukin-2 receptor alpha (*CD25* antigen)

Bold values indicate statistical significance (α < 0.050)

### Histopathological data and TILs

The examined tumors were determined by a qualified pathologist based on the grade of differentiation. The grading was performed in line with the World Health Organization Classification of Tumors of the Digestive System [39]. The tumors were divided into well differentiated (grade 1), moderately differentiated (grade 2) and poorly differentiated lesions. None of the evaluated subgroups showed a statistically significant relation to TILs. Analysis of the other clinical data revealed a significant, weak positive correlation between only patient age and stromal CD45^+^ TILs (r = 0.228, p = 0.045) (Table 2). Tumor budding showed a positive correlation with the tumor size (r = 0.243, p = 0.033) and tumor grade (r = 0.251, p = 0.027). There was a significant difference between tumor budding and tumor grade (p = 0.0363). There were no statistically significant differences between tumor budding and TILs. The distribution of TILs increased with the degree of CRC proliferation (Fig. 4).

### The expression level of CAF markers was increased in colorectal adenocarcinomas

Positive immunohistochemical reactions against α-SMA were highly pronounced in the tumor stroma. The intensity of the observed reactions was higher in cancer tissue than in normal surgical margins. Residual weak expression was also noted in single tumor cells; however, a strong positive response was only noted in the tumor stroma. In the normal colonic glands, weak expression was noted mostly in the lamina propria. In adenocarcinoma, there was a repetitive tendency for a stronger reaction near clusters of cancer cells. A significantly higher level of α-SMA expression was noted in cancer than in normal control (*p* < 0.0001).

Fibronectin was strongly expressed in tumor cells. In an overwhelming number of cases, weaker positive reactions were present in the stroma than in the tumor nests. Positive membrane reactions to fibronectin were observed on inflammatory cells in both normal controls and CRC cases. The level of expression of this marker was not significantly different between normal epithelium and cancer cells (p = 0.3079); however, significant differences were noted in the expression between the normal control and tumor stroma (*p* = 0.0004).

The release of thrombin was particularly present in the range of tumor cells as positive point reactions in individual CRC cells. Thrombin expression was also visible in the tumor stroma; nevertheless, the clusters of cancer cells remained the main compartment for the distribution of positive reactions. Significant differences in thrombin expression were noted between the normal colonic mucosa and CRC tissue (p = 0.003), as well as tumor cells and normal colonic glands (p < 0.001).

The level of FAP-α expression was significantly higher in the tumor stroma than in normal colonic mucosa samples. Moreover, there is a strong positive correlation between FAP-α and FN expression for both tumor stroma (r = 0.491, *p* < 0.0001) and cancer cells (r = 0.453, *p* < 0.0001).

**Fig. 5:**
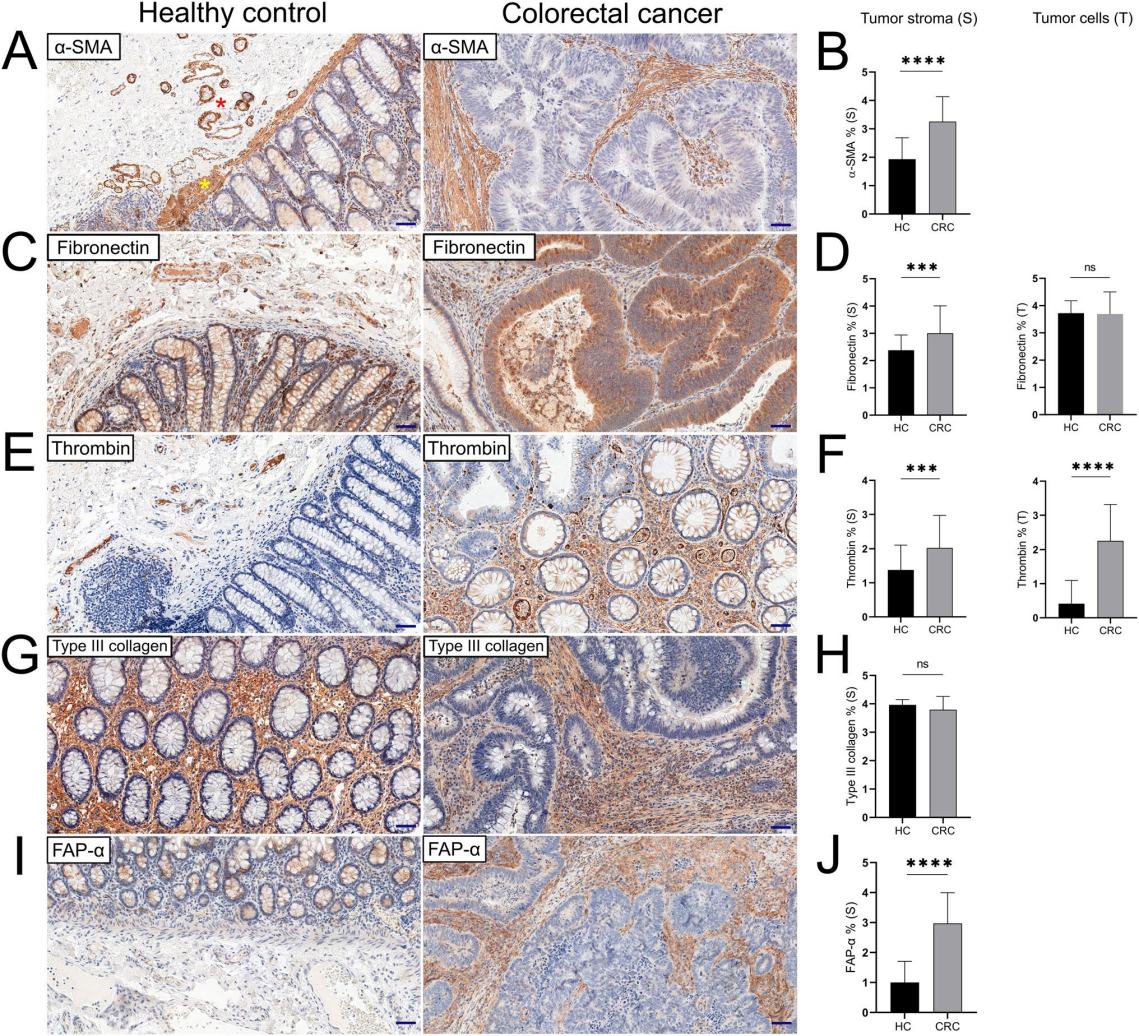
Immunohistochemical reactions for normal and tumoral CAF markers showing differences between healthy colonic mucosa and adenocarcinomas in CRC patients (**a, c**, **e**, **g, i**). The expression pattern of α-SMA (**a**, **b**), collagen (**g**, **h**), and FAP-α (**i**, **j**) was limited to connective tissue; therefore, only the tumor stroma and the area of *lamina propria* surrounding the intestinal glands of colonic mucosa were considered in the estimation (S). α-SMA is a natural biomarker of smooth muscle and endothelial cells, thus the vessels (*red asterix*), muscularis mucosae (*yellow asterix*) and muscularis externa were excluded from the assessment (**a**). Positive reactions against fibronectin (**c**, **d**) and thrombin (**e**, **f**) were also present in epithelial and tumor cells that was taken into consideration in independent measurements (T), not significant (ns); p < 0.001 (***); p < 0.0001 (****). Scale bar = 50 μm

Type III collagen showed specific reactions in the tumor stroma and in the normal connective tissue filling the spaces between the intestinal glands. Despite a similar distribution of reactions between the cancer and normal mucosa (*p* = 0.0583), lower mean values for immunohistochemical expression were noted in cancer (mean: 3.792; SD: 0.4684) than in controls (mean: 3.966; SD: 0.1857), and the visible intensity of positive reactions was higher in the normal control than in the adenocarcinoma tissue. Examples of immunohistochemical reactions together with the obtained results are presented in Fig. 5

### The distribution of TILs in CRC depends on CAFs expression

The expression of α-SMA antigen in colorectal cancer showed a negative correlation with CD8^+^ (r = -0.239, p = 0.0350) and CD45^+^ cells (r = -0.354, p = 0.00146). On the other hand, a positive correlation was found between fibronectin and both stromal CD3^+^ cells (r = 0.336, p = 0.003) and for the intratumoral CD3^+^ cells (r = 0.336, p = 0.0026). A positive correlation was also found in CRC between FN and CD4^+^ cells (r = 0.369, p = 0.001). There was a positive correlation for all CD45^+^ cells but only in the tumor stroma (r = 0.321, p = 0.00421). The expression of FAP-α showed a positive correlation with CD3^+^ (r = 0.465, p < 0.0001), CD4^+^ (r = 0.392, p = 0.001) and CD45^+^ cells (r = 0.494, p < 0.0001).

### The associations between CAF phenotype and clinicopathological parameters

Surprisingly, we found a strong positive correlation between fibronectin and Ki-67 expression in clusters of tumor cells (r = 0.5195, p < 0,0001). A strong positive correlation was also found between the stromal FN expression and tumoral Ki-67 expression (r = 0.3339, p = 0.0028). Stromal and tumoral fibronectin expression increased with the grade of tumor proliferation. A similar trend was found for α-SMA, although in this case the highest level of expression was noted in lesions classified as moderately proliferating (Fig. 4). There was a positive correlation between FAP-α expression and Ki-67 antigen (r = 0.263, p = 0.022). Moreover, the highest level of FAP-α expression was present in highly proliferative lesions (p = 0.0461). The exact results are presented in table (Table 3).

**Table 3:** Associations between CAF phenotype, TILs and clinicopathological data of patients with CRC

Variables	Cancer-associated fibroblasts (CAFs) phenotype						
	Expression pattern						
	Stromal					Tumoral	
	Biomarker						
	α-SMA	Fibronectin	FAP-α	Thrombin	Type III Collagen	Fibronectin	Thrombin
TILs							
CD3^+^ cells	*r* = 0.077	*r* = 0.336	*r* = 0.204	*r* = −0.074	*r* = −0.062	*r* = 0.331	*r* = −0.004
	*p* = 0.504	*p* = 0.0026	*p* < 0.0001	*p* = 0.520	*p* = 0.591	*p* = 0.003	*p* = 0.974
CD4^+^ cells	*r* = −0.031	*r* = 0.374	*r* = 0.392	*r* = 0.167	*r* = 0.046	*r* = 0.369	*r* = 0.115
	*p* = 0.788	*p* = 0.001	*p* = 0.001	*p* = 0.148	*p* = 0.690	*p* = 0.001	*p* = 0.320
CD8^+^ cells	*r* = −0.239	*r* = 0.047	*r* = 0.204	*r* = 0.158	*r* = 0.031	*r* = 0.124	*r* = 0.044
	*p* = 0.035	*r* = 0.686	*p* = 0.077	*p* = 0.171	*p* = 0.790	*p* = 0.277	*p* = 0.701
CD45^+^ cells	*r* = −0.354	*r* = 0.321	*r* = 0.494	*r* = 0.180	*r* = 0.072	*r* = 0.166	*r* = 0.038
	*p* = 0.001	*p* = 0.004	*p* < 0.0001	*p* = 0.117	*p* = 0.536	*p* = 0.147	*p* = 0.746
Immuno regulators							
Stromal							
PD-L1	*r* = 0.081	*r* = 0.357	*r* = 0.541	*r* = −0.021	*r* = 0.096	*r* = 0.202	*r* = −0.099
	*p* = 0.486	*p* = 0.001	*p* < 0.0001	*p* = 0.854	*p* = 0.406	*p* = 0.078	*p* = 0.390
CD14	*r* = 0.168	*r* = 0.160	*r* = 0.314	*r* = 0.072	*r* = 0.065	*r* = 0.158	*r* = −0.093
	*r* = 0.143	*p* = 0.143	*p* = 0.006	*p* = 0.536	*p* = 0.573	*p* = 0.171	*p* = 0.419
CD33	*r* = 0.141	*r* = 0.457	*r* = 0.476	*r* = −0.151	*r* = −0.021	*r* = 0.305	*r* = 0.083
	*p* = 0.220	*p* < 0.0001	*p* < 0.0001	*p* = 0.190	*p* = 0.857	*p* = 0.007	*p* = 0.475
IL-2Rα	*r* = −0.078	*r* = 0.059	*r* = 0.450	*r* = 0.075	*r* = −0.035	*r* = 0.130	*r* = 0.059
	*p* = 0.500	*p* = 0.612	*p* < 0.0001	*p* = 0.519	*p* = 0.763	*p* = 0.258	*p* = 0.611
Tumoral							
PD-L1	*r* = 0.112	*r* = 0.366	*r* = 0.464	*r* = −0.077	*r* = 0.219	*r* = 0.252	*r* = 0.148
	*p* = 0.330	*p* = 0.01	*p* < 0.0001	*p* = 0. 506	*r* = 0.1485	*p* = 0.027	*p* = 0.200
CD14	*r* = −0.046	*r* = 0.108	*r* = 0.039	*r* = 0.095	*r* = 0.069	*r* = 0.186	*r* =−0.057
	*p* = 0.692	*p* = 0.348	*p* = 0.736	*p* = 0.413	*p* = 0.549	*p* = 0.105	*p* = 0.624
CD33	*r* = 0.234	*r* = 0.365	*r* = 0.319	*r* = −0.059	*r* = 0.033	*r* = 0.338	*r* = 0.221
	*p* = 0.039	*p* = 0.001	*p* = 0.005	*p* = 0.053	*p* = 0.774	*p* = 0.002	*p* = 0. 053
IL-2Rα	*r* = 0.113	*r* = −0.076	*r* = 0.307	*r* = 0. 078	*r* = 0.133	*r* =−0.003	*r* = 0.081
	*p* = 0.509	*p* = 0.327	*p* = 0.007	*p* = 0.499	*p* = 0.247	*p* = 0.979	*p* = 0.059
Demographic data							
Age	*r* = -0.218	*r* = 0.006	*r* = 0.145	*r* = -0.112	*r* = -0.092	*r* = -0.041	*r* = 0.021
	*p* = 0.055	*p* = 0.959	*p* = 0.210	*p* = 0.332	*p* = 0.426	*p* = 0.721	*p* = 0.853
Sex (Female/Male)	*p* = 0.4847	*p* = 0.1273	p = 0.0916	*p* = 0.6693	*p* = 0.2266	*p* = 0.6307	*p* = 0.9096
Anatomical site of primary CRC							
Left-sided	*p* = 0.4140	*p* = 0.1841	*p* = 0.2181	*p* = 0.7693	*p* = 0.5493	*p* = 0.1408	*p* = 0.3216
Right-sided							
Histopathology							
Mucinous	*p* = 0.8378	*p* = 0.5338	*p* = 0.5762	*p* = 0.6634	*p* = 0.4138	*p* = 0.1444	*p* = 0.0771
Non-mucinous							
Macroscopic subtype							
Ulcerated	*p* = 0.5649	*p* = 0.6634	*p* = 0.1318	*p* = 0.6822	*p* = 0.0145	*p* = 0.9599	*p* = 0.1608
Non-ulcerated							
Tumor size	*p* = 0.7887	*p* = 0.9465	*p* = 0.5271	*p* = 0.4142	*p* = 0.7297	*p* = 0.3786	*p* = 0.6877
≤ 2 cm & > 2 cm							
Tumor budding	*p* = 0.7965	*p* = 0.4795	*p* = 0.6747	*p* = 0.0645	*p* = 0.3217	*p* = 0.3129	*p* = 0.3540

### The expression levels of CD14, IL-2Rα, CD33 and PD-L1 were disturbed in primary colon adenocarcinomas and positively correlated with TILs

**Fig. 6:**
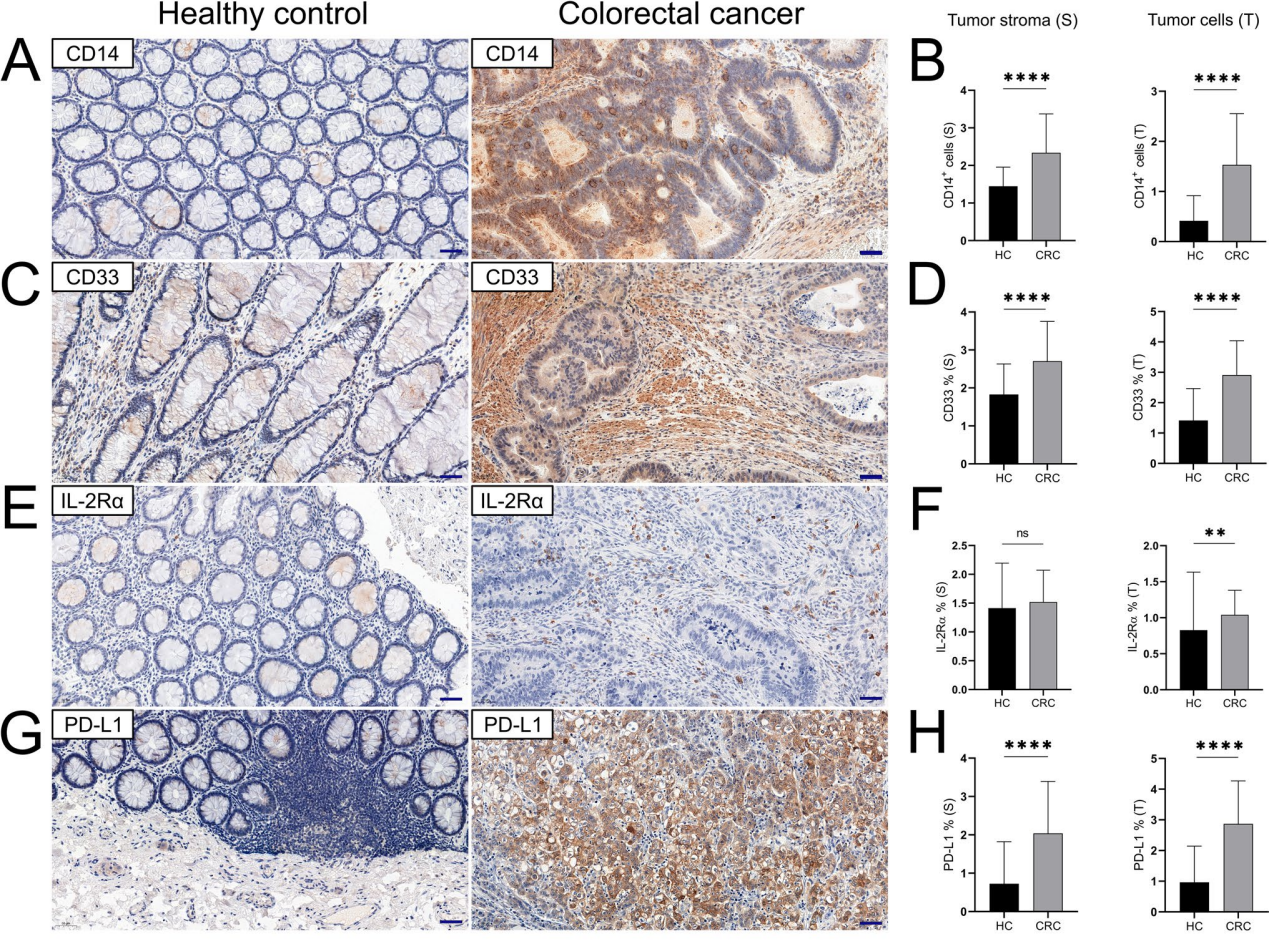
Overexpression of immunoregulatory molecules in colorectal adenocarcinomas compared with normal colonic mucosa (**a, c, e, g**). Reactions for the relevant immune markers were compared between tumor cells and epithelial cells (T) as well as between lamina propria and tumor stroma (S) as shown in graphs (**b**, **d**, **f**, **h**); not significant (ns); *p* < 0.05 (*); *p* < 0.01 (**); *p* < 0.001 (***); *p* < 0.0001 (****). Scale bars represent 50 μm

The number of CD14^+^ cells was significantly higher in cancer tissue than in normal control for both inflammatory infiltrates (p < 0.0001) and the stroma (p < 0.0001) (Fig. 6a, b). IL-2Rα expression was significantly higher in CRC tissue than in normal colonic mucosa (p = 0.0069) and was not significantly different in the tumor stroma (p = 0.3234) (Fig. 6e, f). The level of CD14 expression in CRC increased with tumor budding and was the highest for the Bd3 score (p = 0.0324). The number of CD14^+^ cells was also dependent on the TNM stage (p = 0.0444). Both markers were correlated positively with TILs. A positive correlation was found between tumoral IL-2Rα expression both CD8 (r = 0.481, p < 0.0001) and CD45 antigen (r = 0.378, p = 0.00071). Immunohistochemical expression of IL-2Rα in the tumor stroma showed a positive correlation with CD3^+^ (r = 0.245, p = 0.032), CD8^+^ (r = 0.256, p = 0.025) and CD45^+^ cells (r = 0.331, p = 0.003). The number of CD14^+^ cells in the stroma was positively correlated with CD3^+^ (r = 0.243, p = 0.033) and CD4^+^ cells (r = 0.389, p < 0.0001). PD-L1 expression in CRC was higher than that in healthy colonic mucosa for both the tumor stroma (p < 0.0001) and cancer cells (p < 0.0001) (fig. 6h). Positive reactions against PD-L1 were most often observed in cancer cells usually in the form of a membrane reaction, often accompanied by cytoplasmic colocation (Fig. 6g). Moreover, in many cases, positive staining was noted in the tumor stroma and the distribution of these reactions was likely to correspond to fibroblasts. There was a positive correlation between PD-L1^+^ cancer cells and both CD3 (r = 0.264, p = 0.02) and CD4 (r = 0.408, p = 0.0002) antigens. The stromal expression level was significantly correlated with CD3^+^ (r = 0.320, p = 0.0045) and CD4^+^ (r = 0.316, p = 0.0002) stromal cells. Regarding the CD33 antigen, significant differences in expression were detected between the normal control and CRC samples for both cancer cells and the tumor stroma (p < 0.0001, respectively) (Fig. 6d). In the case of CD33 expression, the membrane type of immunohistochemical reaction was most frequently observed; however, membrane-cytoplasmic expression was also commonly noted (Fig. 6c). Clear cytoplasmic expression of that antigen was present with much less frequency. As with PD-L1 expression, a positive correlation with CD3^+^ TILs was detected for both stromal CD33^+^ cells (r = 0.292, p = 0.0096) and CD33^+^ tumor cells (r = 0.327, p = 0.003). There was a negative correlation between stromal CD33^+^ cells and CD14 expression in the tumor stroma (r = -0.303, p = 0.0073). Moreover, a similar tendency was observed for both of these antigens in intratumoral infiltrates (r = -0.385, p = 0.001).

### Well-differentiated colorectal adenocarcinomas highly expressed immunosuppressive molecules

**Fig. 7:**
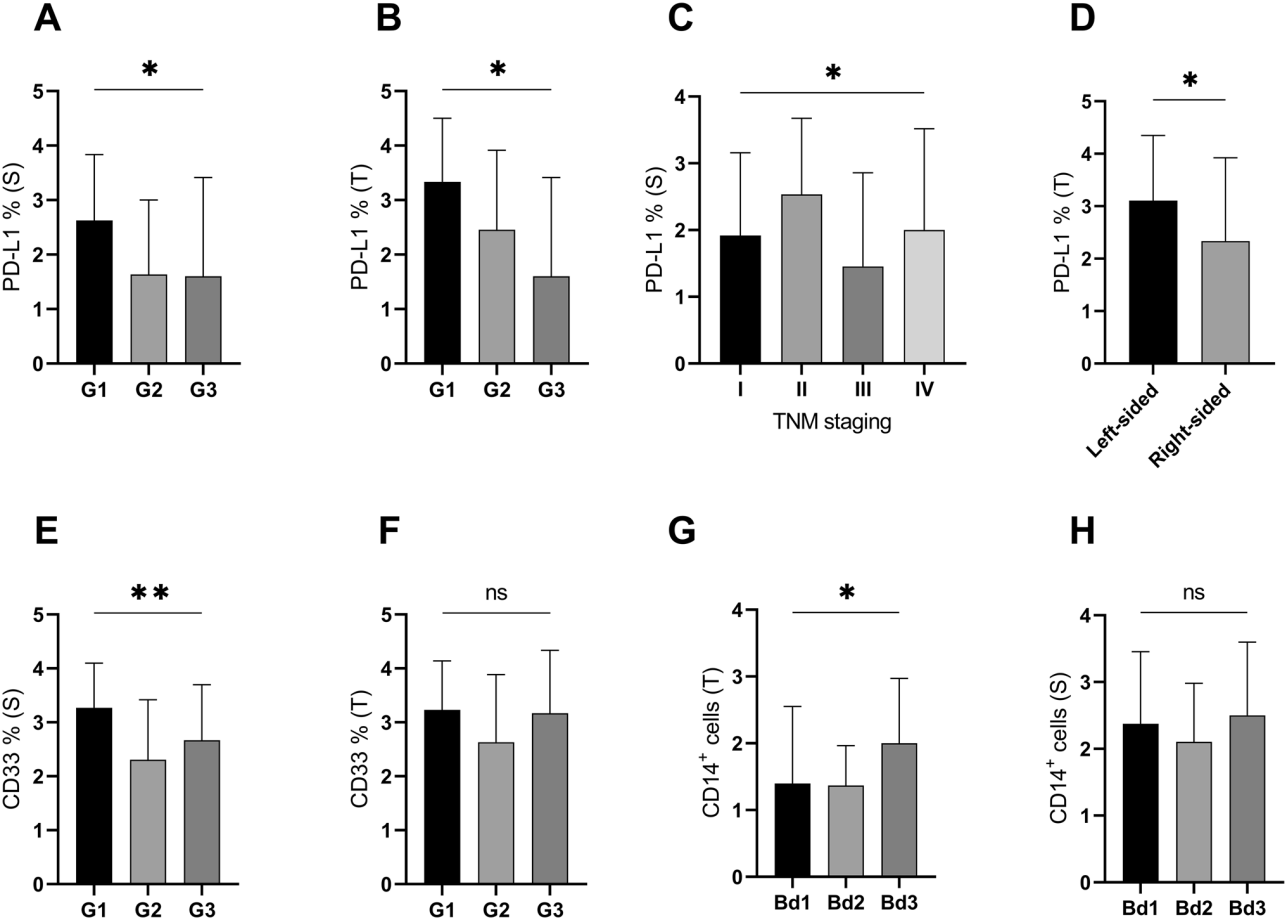
Stromal (S) and tumoral (T) expression of PD-L1 (**a–d**) and CD33 (**e,f**) in human colorectal adenocarcinomas with varying differentiation grades (G1-G3). The expression of PD-L1 in tumor (T) was more pronounced for left-sided lesions than in right-sided (**d**). Stromal expression of PD-L1 differed depending of the tumor stage (**c**). Tumor budding (Bd1–Bd3) affected tumoral expression of CD14 antigen with no impact on its stromal distribution (**g,h**); not significant (ns); *p* < 0.05 (*); *p* < 0.01 (**)

Stromal and tumoral PD-L1 expression depended on the tumor grade and was highest in well-differentiated cancer lesions. The expression level of this ligand in both tissue compartments was significantly decreased in poorly differentiated lesions (Fig. 7a, b). The stromal distribution of PD-L1 was also dependent on the TNM stage, it was highest in stage II and significantly lower for more advanced stages (Fig. 7 c). Well-differentiated adenocarcinomas were also characterized by significantly higher coexpression of the CD33 antigen in the tumor stroma (Fig. 7E).

### CAF markers and immunoregulatory molecules

There was a positive correlation between PD-L1^+^ cancer cells and fibronectin expression in CRC (r = 0.252, p = 0.027). Surprisingly, the positive correlation between the stromal distribution of fibronectin and PD-L1^+^ tumor cells was even more pronounced (r = 0.366, p = 0.001). In the tumor stroma, coexpression of these markers was also evident (r = 0.357, p = 0.001). Stromal CD33^+^ cells significantly correlated with fibronectin in the tumor stroma (r = 0.457, p < 0.0001). A positive correlation was also noted between stromal CD33^+^ cells and FN^+^ tumor cells (r = 0.305, p = 0.007). The expression level of FAP-α positively correlated with stromal (r = 0.541, p < 0.0001) and tumoral PD-L1 (r = 0.464, p < 0.0001), stromal (r = 0.476, p < 0.0001) and tumoral CD33 (r = 0.319, p = 0.005), stromal CD14^+^ cells (r = 0.314, p = 0.006) and stromal (r = 0.450, p < 0.0001) and tumoral IL-2Rα expression (r = 0.307, p = 0.007).

### Clinicopathological characteristics of the colorectal cancer cohort and their associations with immunoregulators

Regarding the demographic data, a significant positive correlation was demonstrated between the patient’s age and IL-2Rα expression for both stromal (r = 0.354, p = 0.002) and tumoral (r = 0.273, p = 0.016) patterns. This variable did not affect the expression of the other markers. The patient’s sex had no influence on the expression of the tested parameters. The histopathological subtype of the primary tumor as well as its macroscopic features had no effect on the expression level of the examined biomarkers. The anatomical location of the primary CRC tumor was of importance for PD-L1 expression but only in the tumor (p = 0.0486) and was higher for right-sided lesions (Fig. 7c). Stromal PD-L1 expression was not significantly different according to the anatomical site (p = 0.3252, Mann–Whitney U test).

## Discussion

Colorectal cancer is a neoplasm in which inflammation plays an important prognostic role. The molecular parameters of TILs are gaining attention as a category of new biomarkers with diagnostic and prognostic significance. Regarding CRC, the prognostically favorable TIL immunophenotype is usually associated with the expression of cytotoxic T cell markers [19, 40], which has been well documented. The adverse effect on the prognostic value in CRC seems to depend on the suppressive impact of the tumor on the regulation of the immune response, especially in relation to T cells [18, 41, 42]. This tendency is confirmed by the beneficial effect of a high TIL density on the prognosis [20, 43]. In our research, we found a significantly lower intensity of inflammatory infiltrations in CRC than in normal control, which seems to confirm the hypothesis of tumor-induced cellular anergy. Due to reports of the suppressive activity of cellular senescence on the immune system [2], to eliminate this factor, we conducted an additional analysis on matched CRC tissue fragments and normal colonic mucosa samples taken from the individual patients, confirming previously obtained results with similar statistical significance. Moreover, in our analysis, we found a weak, positive impact of the patient's age on the number of CD45^+^ cells. Such a conclusion applies only to quantitative assessments and obviously does not exclude changes in inflammatory cell activity, which occur during the cellular senescence of inflammatory cells and may adversely affect the immune response [41]. Contrary to this statement, we also noted a significant effect of patient age on the level of IL-2Rα expression in both tumor and its stroma. The obtained result may indicate an increasing Treg population as an effect of cellular senescence, which confirms the suppressive effect of aging on T cells [41].

Another important conclusion resulting from our work is the positive coexpression of TIL markers and the Ki-67 antigen. This marker is described as a favorable prognostic parameter in CRC [25, 26]. Given the stronger positive correlation of the coexpression of CD4 and Ki-67, we suppose that this marker may have a higher positive prognostic value than the previously mentioned CD3 and CD8 antigens. This fact also seems to confirm the negative correlation of TILs with the TNM stage; in our study, this relationship was most strongly exposed for stromal CD4^+^ TILs. Furthermore, significantly more CD4^+^ T cells were found in early stage I CRC than in the more clinically advanced stages of CRC. The obtained conclusions should be considered with great caution, because a higher Ki-67 index is not always interpreted as a favorable marker in CRC [24]. Our research is also limited by the fact that immunohistochemical analyses of the coexpression of selected TIL markers were conducted only in relation to selected clinicopathological data, and the survival curves of the treated patients were not analyzed. These hypotheses require further research in a larger group of patients, including a survival analysis 

The colorectal adenocarcinomas examined in this study seem to directly induce suppression of the host immune response by the overexpression of PD-L1 and CD33 molecules. The expression of these immunosuppressants in CRC was predominantly pronounced for well-differentiated lesions. This observation suggests an increase in immunosuppressive effects in the early stages of tumor development, which seems to confirm the obtained significance between the TNM stage and PD-L1 expression. This position is additionally supported by the results obtained for the distribution of CD3^+^ and CD4^+^ cells, the number of which was the highest in early TNM stages and constitutively decreased with clinical advancement of the tumor. We also found strong positive correlations between these subtypes of T cells and PD-L1 expression. With this in mind, we believe that monitoring immunosuppressive agents has the potential to aid early CRC screening, which requires further studies regarding measurement of the level of PD-L1 and other immunosuppressive factors.

Our results regarding the expression of CAF markers suggest an important role of intratumoral fibrosis as an indirect mechanism of suppression of the immune response in CRC. Intestinal fibrosis often coexists with chronic inflammation, which is especially common during inflammatory bowel disease (IBD) [29]. In CRC, persistent inflammation is one of the pathognomonic hallmarks of this tumor; however, the importance of fibrosis in this type of cancer and its relation to TILs have not been widely studied.

In this study, we found that the overexpression of CAF markers in colorectal adenocarcinomas was associated with TILs. The expression of α-SMA and collagen III was essentially present in the tumor stroma and compared with the healthy intestinal mucosa it was limited to the connective tissue surrounding the colonic glands. We expected increased collagen expression in CRC, rather than in the control mucosa, due to the overactivity of CAFs leading to the increased release of this fibril protein in cancer. However, the obtained differences regarding its expression were not significantly different; moreover, the mean values ​​of the percentage of collagen distribution in CRC were decreased compared with those in the normal controls and the intensity of the observed positive reactions turned out to be more pronounced in the normal colonic mucosa than in the tumor itself. Inflammatory colorectal polyps showed high expression of metalloproteinases (MMPs) with proteolytic activity related to degradation of the extracellular matrix [44], and in CRC, the activity of metalloproteinase-9 was increased and associated with the promotion of distant metastases [45]. The potential catalytic overactivity of these enzymes in cancer may explain the degradation of the collagen structure, which in turn explains the differences that were observed.

On the other hand, the expression of thrombin and fibronectin was particularly present within clusters of cancer cells. Moreover, a positive membrane reaction for fibronectin was also noted within inflammatory cells in both colonic mucosa and cancer samples. It is surprising, however, that some fibronectin isoforms are actively released by inflammatory cells during infection, and the mechanism itself serves to intensify the phagocytosis process to combat the current source of infection, which is mediated by the activation of avβ3 integrin [46]. We suppose that the observed positive membrane reaction to fibronectin in inflammatory cells surrounding CRC may serve to induce and intensify tumor cell phagocytosis by immune cells of the phagocyte system. This hypothesis may explain the positive correlation found between TILs and FN.

Contrary to this statement, we found that activation of CAFs enhances the release of fibronectin in both tumor cells and TME. In CRC, however, the activation of avβ3 by fibronectin may have a completely different biological outcome than during infection, which results in tumor cell growth and causes drug resistance that ultimately leads to the progression of colorectal cancer [47]. In gastrointestinal tumors, the expression level of avβ3 was highly increased [48] and the activation of this integrin enhances the proliferation of cancer cells and increases the release of PD-L1, which is one of the mechanisms of cancer immune evasion [49, 50]. PD-L1 overexpression in fibroblasts often takes place during increased fibrosis [51], which in the case of cancer development may emphasize the negative influence of CAFs on the phagocytic abilities of inflammatory cells acting in the stroma. To date, the immunosuppressive impact of CAFs on T cell activity has been confirmed in head and neck squamous cell carcinoma [52]. In our study, the activation of CAFs seems to influence the intensity of inflammatory infiltrates in CRC tissue. We found a strong positive coexpression of TIL markers and stromal FAP-α. Our analyses also showed that the activation of CAFs enhances the phenomenon of immunosuppression in colorectal adenocarcinomas, which is confirmed by the highly positive correlations between FAP-α and selected immune markers. Moreover, we confirmed a strong positive correlation of fibronectin with Ki-67 proliferation antigen and we noted that PD-L1 was also present in the tumor stroma, which may suggest an important role of FN in inducing CRC progression and stromal PD-L1 expression via CAFs. The last hypothesis is supported by the strong positive correlation between PD-L1 and FN that we observed in our research. Moreover, the increased release of PD-L1 by fibroblasts may also occur under the influence of TGF-β, which stimulates these cells to transport this ligand in extracellular vesicles (EVs) [53]. In one of our recent studies we proved that in CRC, there is an abundant release of EVs, and the expression level of exosome markers was significantly different according to the N stage [54].

The positive membrane reaction to fibronectin on inflammatory cells may also suggest the influence of thrombin on the release of FN by these cells, which is important in remodeling the tumor microenvironment. Thrombin increases the release of fibronectin by MDSCs at both the mRNA and protein levels [55], and the catalytic activity of thrombin leads to the release of the mature form of TGF-β1 from platelets, resulting in cancer immune evasion [56]. Thrombin through TGF-β also leads to the release of fibronectin from epithelial cells [57], which, in relation to our results, may indicate a feedback effect of thrombin on FN release directly from cancer cells. Thrombin may also exhibit immunosuppressive and profibrotic properties by changing the polarity of macrophages toward profibrotic M2a phenotype and by increasing fibroblast proliferation and differentiation of fibrocytes [58].

The concept of the immunosuppressive role of platelets in CRC is consistent with current reports. Guinney et al. proposed the division of CRC into four consensus molecular subtypes (CMSs), the CMS4 subtype corresponds to the mesenchymal phenotype of this tumor, the pathogenesis of which involves ECM remodeling signaling pathways [59]. In our work, we observed a weaker intensity of positive reactions against collagen in the tumor stroma, which may emphasize MMP overexpression. The degradation of the ECM by metalloproteinases and the mediators released by platelets that affect the vessel walls allow them to be extravasated into the tumor microenvironment. Platelets also have the potential to regulate the cellular response by directly interacting with immunologically competent cells as well as by influencing the secretion of cytokines such as TGF-β, and other immunoregulators. Moreover, their importance is underlined in the pathogenesis of CRC of the CMS4 subtype [60].

**Fig. 8:**
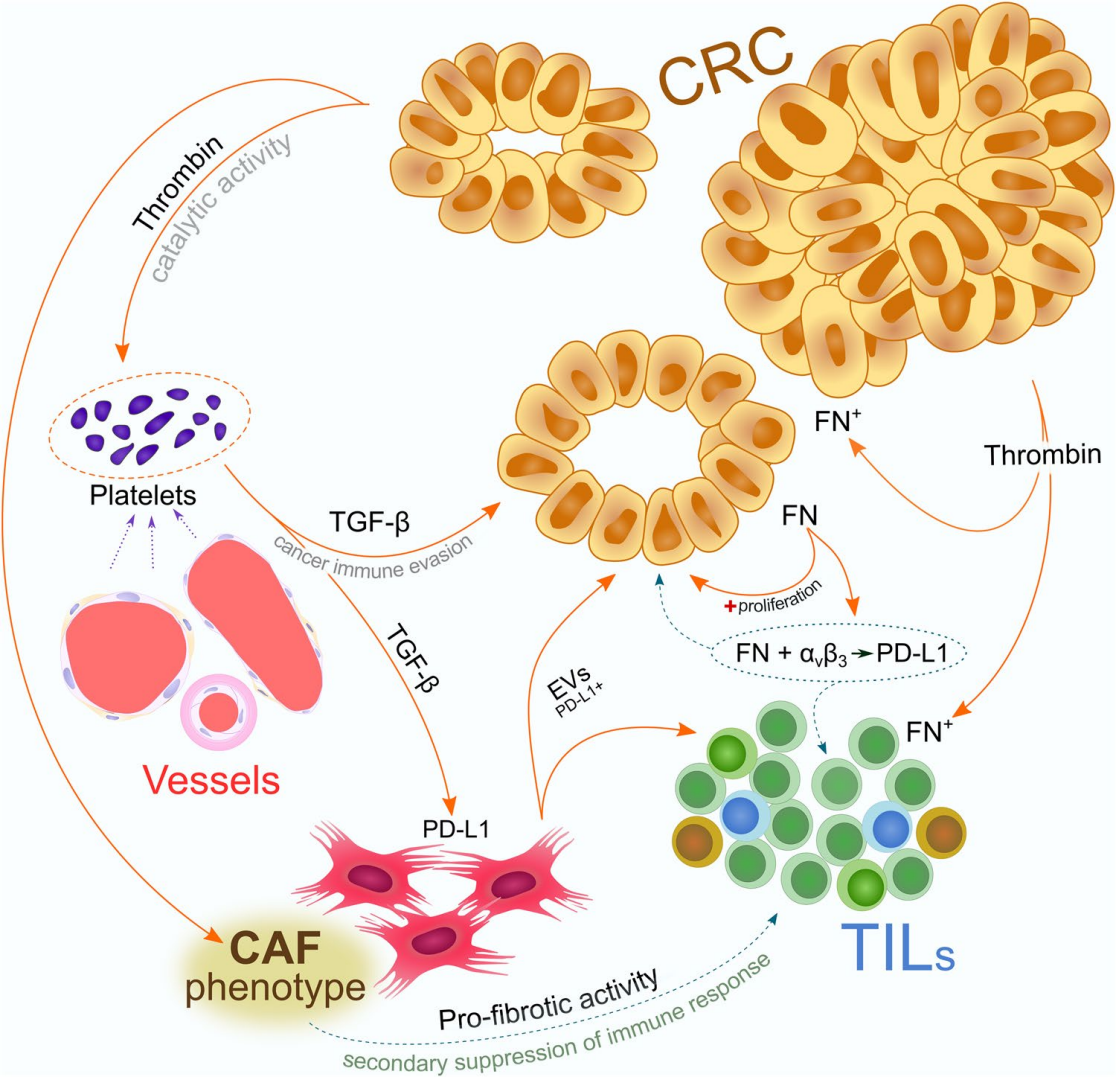
Proposed theoretical model linking cancer induced fibrosis with secondary suppression of host immune response. Profibrotic activity of colorectal cancer (CRC) leads to the activation of cancer-associated fibroblast (CAFs) and then regulate the tumor immunity. Excessive release of thrombin and fibronectin (FN) by cancer cells changes the tumor microenvironment, which promotes disease progression. Thrombin is responsible for the aggregation of platelets from vascular beds and its catalytic activity releases the mature form of TGF-β from the platelets. The biological activity of thrombin leads to the release of fibronectin (FN^+^) from cancer and inflammatory cells and directly enhances the fibroblast proliferation and fibrocyte differentiation, which promotes CAF phenotype. The TGF-β released by platelets has an immunosuppressive activity and additionally stimulates the production of PD-L1 in CAFs; this ligand is further transported in extracellular vesicles (EVs) and then, incorporated in its active form by endocytosis into donor cells, including cellular component of the tumor stroma and cancer cells. Increased proliferation and activity of stromal CAFs reduce the access of tumor infiltrating lymphocytes (TILs) to tumor cells and the suppressive activity of CAFs by releasing PD-L1 in EVs that directly affects T cells. Thrombin also acts on TILs leading to the release of fibronectin by the immune cells and overexpression of FN increases the release of PD-L1 and shows a proliferative effect on CRC dependent on the activation of α_v_β_3_ integrin

In view of the conclusions resulting from our study and the latest literature review, we present on the prepared figure our theoretical concept of the importance of CAFs in CRC and its apparent role in tumor immunity (Fig. 8). The immunosuppressive activity of CRC in our study is also supported by the significantly higher expression of PD-L1 and CD33 in cancer than in normal control. The positive correlation of these suppressive regulators of the immune response with CD3 and CD4 cells emphasizes the importance of T cells in CRC. On the other hand, our results regarding the expression of CD14 antigen highlight an important role of macrophages or dendritic cells in the pathogenesis of CRC. CD14 is also a marker of monocytic myeloid-derived suppressor cells (M-MDSCs), and its expression may suggest increased distribution of this MDSC subpopulation in the tumor milieu [61]. The increased number of M-MDSCs in colorectal cancer may be the result of the activity of CAFs, which facilitate the migration of monocytes from the peripheral blood to the TME [62]. Finally, an accurate comparison of inflammatory infiltrates in matched pairs of healthy colonic mucosa and CRC samples confirmed the reduced distribution of immune cells in the tumor.
